# Editorial: New insights into the role of imaging in large vessel vasculitis

**DOI:** 10.3389/fmed.2024.1528452

**Published:** 2024-12-03

**Authors:** Juan Molina-Collada, Philipp Bosch, Eugenio de Miguel, Wolfgang A. Schmidt, Christian Dejaco

**Affiliations:** ^1^Department of Rheumatology, Hospital General Universitario Gregorio Marañón, Instituto de Investigación Sanitaria Gregorio Marañón (IiSGM), Madrid, Spain; ^2^Department of Rheumatology and Immunology, Medical University of Graz, Graz, Austria; ^3^Department of Rheumatology, Hospital Universitario La Paz, Madrid, Spain; ^4^Immanuel Krankenhaus Berlin, Medical Center for Rheumatology Berlin-Buch, Berlin, Germany; ^5^Department of Rheumatology, Hospital of Bruneck (ASAA-SABES), Teaching Hospital of the Paracelsus Medical University, Brunico, Italy

**Keywords:** imaging, ultrasound, MRI, PET-CT, large vessel vasculitis, giant cell arteritis, polymyalgia rheumatica, Takayasu arteritis

Due to the heterogeneity of clinical manifestations, diagnosing, monitoring, and stratifying risk in large vessel vasculitis (LVV) can be challenging. As a result of technological progress, imaging plays an increasing role in the management of LVV. Ultrasound (US), 18-FDG positron emission tomography/computed tomography (PET/CT), magnetic resonance imaging (MRI), and CT have proven diagnostic value and yielded promising data for the assessment of disease activity in giant cell arteritis (GCA) and Takayasu arteritis (TAK) (1). Since the first description of the use of US in GCA in 1995 (2), numerous studies have confirmed the diagnostic value of imaging for LVV, and the latest 2023 EULAR recommendations (3) reinforce the use of imaging for diagnosis, as well as its potential role in monitoring and assessment of vascular damage. Moreover, imaging was included for the first time in the new 2022 ACR/EULAR classification criteria for GCA (4) and TAK (5). Temporal artery (TA) US carries the same weight as TA biopsy for GCA classification, and evidence of vasculitis by imaging is an absolute requirement for the application of the TAK classification criteria.

However, several unmet needs remain, such us investigating the value of imaging composite scores for diagnosing, monitoring and prognosis of LVV, the prognostic value of positive imaging in patients in clinical remission, and the optimal timing for using imaging to detect vessel wall damage. In addition, as technological advances require constant validation of new imaging applications, this field is continuing to evolve. The articles included in the current Research Topic provide new insights and potential applications of imaging in LVV management.

Recently, interest has grown in using US to quantify vascular inflammation in GCA, and several US scores have been proposed for diagnosis and monitoring (6–10). However, they require extensive validation before they can be applied in research and clinical practice (3). In the current Research Topic, Conticini et al. investigated the diagnostic accuracy of three scores [Southend halo score, halo count, and OMERACT GCA US Score (OGUS)] in 79 patients with suspected GCA. All three scores showed good sensitivity (>70%) and excellent specificity (97%). In particular, for OGUS, a threshold of 0.81 could be employed for diagnostic purposes, although this score was primarily developed for monitoring.

Schweiger et al. retrospectively investigated the incidence and predictors [including US determined intima-media thickness (IMT)] of glucocorticoid related side effects in 138 patients with GCA. Chronic kidney disease, fractures, cataracts, dementia, and hypertension were the most frequent events. In multivariable analysis, relapses during follow-up predicted diabetes, likely due to increased glucocorticoid use. However, analytical parameters of inflammation and endothelial dysfunction, including pulse-wave velocity and IMT by US were not linked with adverse events of glucocorticoids.

The diagnosis of GCA by US relies on traditional elementary lesions such us the halo sign (inflammatory concentric thickening of the arterial wall). The halo sign is normally determined on a visual basis applying the OMERACT criteria (11). However, there are ongoing efforts to establish cut-offs for the measurement of the arterial wall thickness (IMT) in different territories for diagnostic and monitoring purposes (12–15). Seitz et al. studied cut-off values and the diagnostic accuracy of IMTs of TA segments measured by US in GCA, using for the first time a dual reference standard, namely clinical diagnosis at the patient level and MRI of the head at the segmental level. Optimal US IMT cut-offs (of both walls measured together with complete compression) were 1.01 mm for the common superficial TA, 0.82 mm for the frontal branch and 0.69 mm for the parietal branch, with 79.7% sensitivity and 90.0% specificity for the diagnosis of GCA. The authors demonstrated further in a sub-analysis that sensitivity and specificity of the cut-offs were lower in high cardiovascular risk patients suggesting that cut-offs might need to be adjusted based on the individual cardiovascular risk profile.

Nielsen et al. presented the protocol for the DANIsh VASculitis cohort (DANIVAS), a national multicenter study aiming to prospectively collect clinical data and biobank material from polymyalgia rheumatica (PMR) and GCA patients. Specific objectives include the evaluation of treatment needs in GCA patients with/without LV involvement, in PMR with/without subclinical GCA, and the prognostic role of imaging for aneurysm formation.

In a groundbreaking study, Skoog et al. evaluated the role of superb microvascular imaging (SMI) to visualize neovascularization in TA and assessed its diagnostic performance alongside US in patients with suspected GCA. SMI detected neovascularization in 14 (43%) of 33 GCA patients, and this finding was associated with more widespread cranial disease and a higher halo count. While SMI did not improve sensitivity or specificity of the exam, it might serve in future as a marker to stratify GCA patients for disease severity.

In this Research Topic, two narrative reviews focusing on the role of PET/CT in the management of LVV are also presented. Collada-Carrasco et al. focused on the use of PET/CT in the diagnosis and follow-up of LVV, including a valuable comparison of the most relevant diagnostic PET/CT scales for LVV and PMR. Thibault et al. examined the value of PET/CT in the diagnosis and follow-up of GCA, including all studies on its role in predicting relapses. Moreover, Ni and Kohler reviewed recent advances in LVV imaging, highlighting the combination of imaging modalities, and newer techniques like contrast-enhanced US, shear wave elastography and ocular US.

Another interesting aspect in this Research Topic is the multicenter retrospective study of patients with vasculitis in Poland (POLVAS) presented by Milchert et al. They demonstrated an increase in GCA diagnosis from 2008 to 2019, reaching 8.38 per 100,000 in patients 50 years or older, which can in part be attributed to the introduction of fast-track diagnostic pathways in several centers.

Petzinna et al. reviewed the pathophysiological role of vascular adhesion protein-1 (VAP-1) in vascular inflammation, focusing on GCA and PMR. They highlighted VAP-1′s involvement in immune cell adhesion, migration, and its enzymatic contributions to oxidative stress and tissue damage, as well as recent imaging advances targeting VAP-1, such as [68Ga]Ga-DOTA-Siglec-9 PET/CT, offering new insights into VAP-1′s role in GCA and PMR pathogenesis.

In summary, this Research Topic provides a collection of articles offering new insights into LVV imaging, and we believe it represents a valuable contribution to a constantly evolving field of research.
